# Different Kinds of Eyes

**Published:** 1874-03

**Authors:** 


### DIFFERENT KINDS OF EYES.

NO BRANCH of science has been more thoroughly mastered than optics. The principle of vision must be essentially the same in all eyes, but they differ remarkably, according to the habits of the animal. Birds of lofty flight, as the condor, eagles, vultures, and carrion seeking prowlers of the feathered race, have telescopic visions, and thus they are enabled to look down and discover their unsuspecting victims. As they approach noiselessly from above the axis of vision changes—shortening, so that they can see as distinctly within one foot of the ground as when at an elevation of one mile in the air.

This fact explains the balancing of a fish-hawk on its pinions half a mile above a still pond, watching for fish. When one is selected, down the savage hunter plunges, the focal axis varying always to the square view of his intended prey. As they ascend the axis is elongated by a curious muscular arrangement, so as to see far off again.

Snails have their keen eyes at the extremity of flexible horns, which they can protrude or draw in at pleasure. By winding the instrument round the edge of a leaf or stalk, they can see how matters stand on the opposite side.

The hammer-headed shark has its wicked looking eyes nearly two feet apart. By will effort they can bend the thin edges of the head, on which the organs are located, so as to examine the two sides of an object the size of a full sized codfish.

Flies have immovable eyes. They stand out from the head like half an apple, exceedingly prominent. Instead of smooth hemispheres they have an immense number of facets, resembling old fashioned glass watch seals, each one directing the light directly to the optic retina. That explains why they cannot be approached in any direction without seeing what is coming.

